# Influence of TEOS/MTMS Binder Composition on the Microstructure and Heat Resistance of Electrophoretic Deposited Alumina–Silica Composite Coatings

**DOI:** 10.3390/mi17080906

**Published:** 2026-07-29

**Authors:** Dohyeon Mun, Jae-Young Bae

**Affiliations:** Department of Chemistry, Keimyung University, 1095 Dalgubeol-daero, Dalseo-gu, Daegu 42601, Republic of Korea; diadieto@naver.com

**Keywords:** electrophoretic deposition, alumina-silica composite, electric vehicle, fire safety, siloxane binder, thermal insulation

## Abstract

As the power and voltage of electric vehicles increase, the thermal stability of battery components, such as busbars, becomes critical. This study proposes a method for inorganic thermal insulation and insulating coating using alumina–silica hybrid particles deposited via electrophoretic deposition (EPD). We investigated the effects of copper substrate pre-treatment and the weight ratio of tetraethylorthosilicate (TEOS) to methyltrimethoxysilane (MTMS) on the quality of the coating. AFM analysis confirmed that a 1-min pre-treatment optimized surface roughness, which helped prevent cracks during the drying process. Among the various compositions tested, the sample with 30% TEOS demonstrated the highest thermal stability (670 °C) and dispersion stability (zeta potential of −15.5 mV), attributed to the increased density of the siloxane network. All samples maintained insulation performance up to 6.3 kV. These findings present an effective strategy for enhancing the fire safety of EV electrode materials.

## 1. Introduction

With the increasing power output and voltage of electric vehicles (EVs), the thermal stability of batteries and power transmission components has become a crucial factor in ensuring vehicle stability [1]. Specifically, busbars connecting batteries and power electronic modules are highly susceptible to secondary accidents if current interruption is delayed during a fire [2,3]. Therefore, there is a need for coating technology that can maintain structural and electrical integrity even in high-temperature environments [4,5]. Epoxy resins are commonly used to protect such electrode materials [6,7,8]. These compounds contain two or more epoxy groups in their structure and react well with various substances, enabling the development of materials with diverse physical properties [9]. There is ongoing research focused on developing materials to protect electrode components by utilizing the characteristics of epoxy systems. For example, researchers are working to synthesize an epoxy powder coating composition with excellent heat resistance and insulating properties by adding isocyanate-modified epoxy resin to bisphenol A-type epoxy resin [10]. Another area of investigation involves the use of liquid crystalline epoxy resins (LCERs), which, unlike conventional epoxy resins with randomly arranged structures, feature regularly aligned molecular structures that can be effectively applied to electrode material coatings [11].

However, a drawback of previously studied epoxy resins is their tendency to develop bubble defects during application, leading to partial discharge and reduced insulation performance [12,13]. Additionally, applying epoxy to busbars with complex shapes presents challenges in terms of processing [14,15]. Electric vehicles are particularly vulnerable to fires in high-voltage batteries, with one notable characteristic of such fires being thermal runaway, which can raise temperatures to over 1000 °C [16,17,18,19].

Pure epoxy begins to thermally decompose around 300 °C, with maximum mass loss occurring between 363–386 °C. This thermal fragility indicates that epoxy resins used alone are unsuitable for certain high-temperature applications. Consequently, it is evident that coatings made solely of epoxy resin have limitations in protecting the electrode materials of electric vehicles. Recently, research has progressed in enhancing heat resistance by utilizing inorganic materials instead of epoxy resins. A representative example is the synthesis of ladder-like polysilsesquioxane (LPSQ) resin and its application as an electrode material [20,21]. However, coating electrode materials with LPSQ resin may lead to issues such as pinholes. Small defects, like pinholes, can cause partial discharge, resulting in thermal, chemical, and physical damage [22,23]. Despite the limitations of existing research, ensuring stability during electric vehicle fires with materials that possess superior heat resistance and insulation properties remains a critical challenge. Recently, there has been growing interest in insulating and heat-resistant materials created using the electrophoretic deposition (EPD) method. EPD involves the movement of charged particles under an electric field to deposit material. This technique immerses electrodes in a solution containing dispersed charged colloidal particles and applies a voltage to form a uniform thin film on the desired substrate, allowing for the formation of consistent and refined coatings even on complex and irregular shapes like busbars. Moreover, the equipment for this process is relatively simple, enabling quick and efficient coating, while the thickness of the deposited thin film can be easily controlled by adjusting the applied voltage, process time, and particle concentration [24,25,26]. In recent years, research has also explored the advantages of the EPD method for vapor deposition with various materials. For instance, studies are underway to utilize mesoporous silica for water vapor adsorption/desorption-related vapor deposition [27,28]. This study proposes an inorganic thermal insulation and insulating coating method using alumina–silica hybrid particles in an EPD coating system that can maintain stable functionality even in high-temperature fire environments. To achieve this, we will confirm deposition stability by pretreating the copper busbar surface and analyze the effects of varying the TEOS weight ratio to MTMS on sol stability, microstructure, thermal and electrical properties, and evaluate its overall performance.

## 2. Materials and Methods

### 2.1. Materials

The following reagents were used as received without further purification:

Methyltrimethoxysilane (MTMS, DAEJUNG, Siheung-si, Republic of Korea) and tetraethyl orthosilicate (TEOS, 98 wt%, Sigma-Aldrich-Korea, Seoul, Republic of Korea) served as siloxane binders. TM-50 silica sol (LUDOX^®^TM-50 colloidal silica, Sigma-Aldrich-Korea, Seoul, Republic of Korea) and PS-30 tabula alumina powder (PS-30 platelet alumina, YKC Corporation, Seoul, Republic of Korea) were the primary inorganic components for the composite coating. For the chemical pre-treatment of the copper substrates, hydrogen peroxide (H_2_O_2_, 30%, DAEJUNG, Siheung-si, Republic of Korea) and sulfuric acid (H_2_SO_4_, 98%, DAEJUNG, Siheung-si, Republic of Korea) were utilized. Isopropyl alcohol (IPA, 99.5%, DAEJUNG, Siheung-si, Republic of Korea), dimethylformamide (DMF, 98%, DAEJUNG, Siheung-si, Republic of Korea), and propionic acid (C_3_H_6_O_2_, DAEJUNG, Siheung-si, Republic of Korea) served as solvents and catalysts for the sol–gel reaction. Ethanol (EtOH, 95%, DAEJUNG, Siheung-si, Republic of Korea) was used for initial substrate cleaning.

### 2.2. Substrate Preparation

Copper electrode materials were subjected to chemical etching to optimize surface adhesion for EPD. The treatment solution was prepared by mixing 30 mL of H_2_O_2_, 30 mL of H_2_SO_4_, and deionized water, stirring the mixture for 30 min. The copper surfaces were first degreased with EtOH and then immersed in the prepared etching solution for 1 min at room temperature. After treatment, the substrates were rinsed and dried to achieve the desired surface roughness (Sq ≈ 1.1706 µm) for mechanical interlocking.

### 2.3. Preparation of Electrophoretic Deposition (EPD) Solutions

The coating solutions were synthesized via a sol–gel process with varying TEOS/MTMS weight ratios (Table 1).

Synthesis was conducted by adding 48 g of isopropyl alcohol (IPA), 4.8 g of dimethylformamide (DMF), and 4 g of propionic acid into a three-necked flask while stirring. The solution was heated to 60 °C, followed by the addition of 165 g of TM-50 silica sol and specific ratios of MTMS and TEOS. Finally, 80 g of PS-30 plate-shaped alumina powder was added and reacted for 30 min to obtain a white EPD solution. Notably, the 20% TEOS sample underwent rapid gelation during synthesis due to the non-linear crosslinking kinetics of TQ silanes at this specific T/Q ratio, rendering it unsuitable for deposition [29]. Consequently, this study focused on comparing the 0%, 10%, and 30% samples [30].

### 2.4. Electrophoretic Deposition Process

The copper substrates were initially cleaned with ethanol (EtOH) and pre-treated by immersion in the copper treatment solution for 1 min. During the electrophoretic deposition (EPD) process, the pre-treated copper sample acted as the anode (+), while an untreated copper sample (washed only with EtOH) served as the cathode (−). Deposition was conducted for 10 min at a voltage of 10 V and a current of 1 A, followed by final curing in an oven at 100 °C.

### 2.5. Characterization

Surface roughness was analyzed using atomic force microscopy (AFM; JPK Instruments, Berlin, Germany, NanoWizard Ultra Speed AFM). Surface and cross-sectional morphologies were observed using a scanning electron microscope (SEM; JEOL, Tokyo, Japan, IT-500) at an accelerating voltage of 10 kV, complemented by energy dispersive spectroscopy (EDS; JEOL, Tokyo, Japan, IT-500) for elemental analysis. Zeta potential measurements were conducted using 1% diluted samples in IPA to evaluate colloid dispersion stability (Malvern Panalytical, Malvern, UK, Zetasizer Nano ZS). Thermogravimetric analysis (TGA; TA Instruments, New Castle, DE, USA, TA-TGA550) was performed from room temperature to 800 °C at a heating rate of 10 °C/min. Fire resistance was tested by exposing fixed samples to direct flames at a distance of 15 cm. Pinhole detection (Paint Holiday Detector, Paint Test Equipment, Congleton, Cheshire, UK, Paint Holiday Detector S400) was performed from 0.5 kV to 6.3 kV in 0.5 kV increments to verify dielectric strength.

## 3. Results

### 3.1. Substrate Surface Analysis

**Figure 1:**
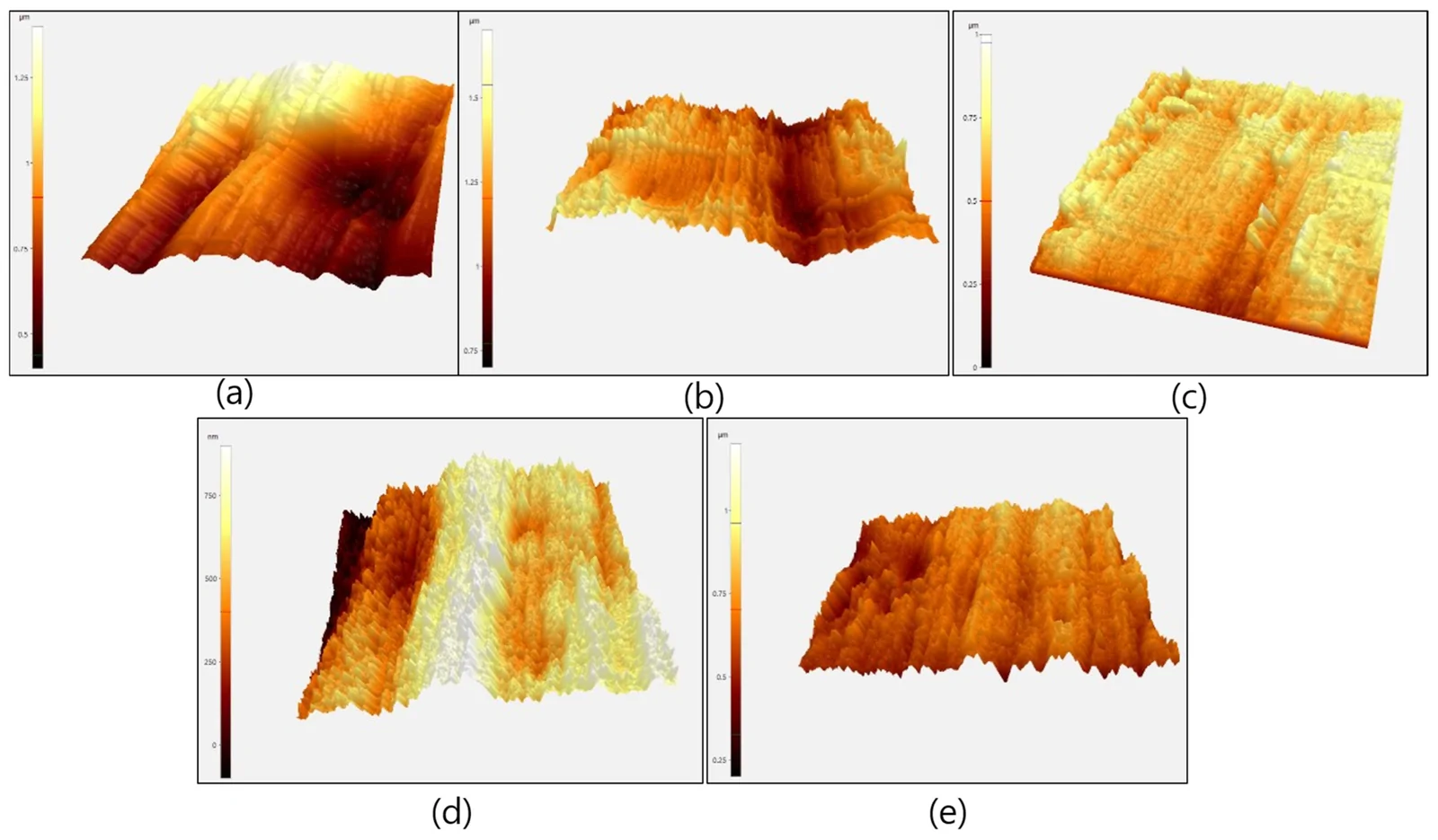
AFM images for verifying sample surface differences due to pretreatment: (**a**) untreated sample; (**b**) 1 min; (**c**) 2 min; (**d**) 30 °C 1 min; (**e**) 50 °C 1 min.

The surface condition of the substrate in the EPD coating directly affects the adhesion of deposited particles and the formation of defects during the drying process. To evaluate this, we analyzed the surface roughness of copper samples with the same nominal liquid composition but different processing times and temperatures using AFM (Figure 1).

AFM analysis results showed that the average roughness of the sample pretreated for 1 min increased compared to the sample without surface pretreatment. This increase in roughness, characterized by the formation of microsurface irregularities, enhanced the effective contact area and consequently improved the overall bonding strength in the EPD deposition method [31]. However, when the surface treatment time was extended to 2 min or conducted under high-temperature conditions (50 °C), surface dissolution and rearrangement became dominant, leading to even faster planarization [31,32] (Table 2).

**Figure 2:**
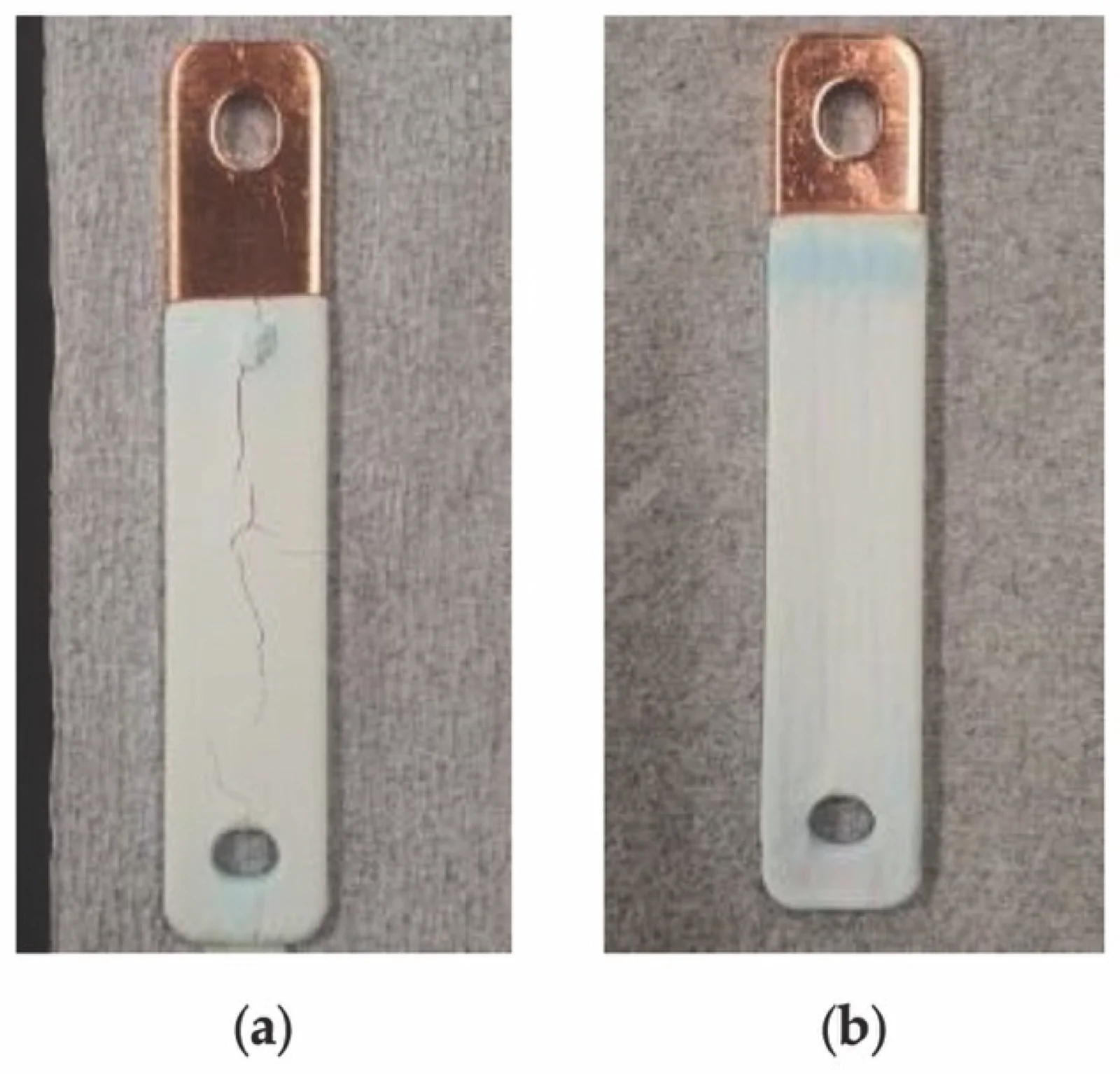
Difference in deposition intensity according to sample pretreatment: (**a**) untreated sample; (**b**) 1 min sample.

These changes in surface roughness directly impact the deposition results. When deposition was carried out under the same EPD conditions (Figure 2), cracks formed in specimens with excessive planarization after drying (Figure 2a), while a dense, crack-free coating layer developed in specimens with appropriate roughness (Figure 2b). This demonstrates that the mechanical interlocking effect between the substrate and particles during the EPD process is crucial for deposition stability.

### 3.2. Colloidal Stability and Zeta Potential Analysis

Zeta potential analysis was conducted by diluting each sample in IPA to a concentration of 1% and repeating the analysis three times (Table 3). It was observed that the absolute value of the zeta potential increased from −3.63 mV to −15.5 mV as the TEOS content increased. Generally, the dispersion stability of colloids increases with a larger absolute value of the zeta potential, and in aqueous solvent systems, +30 mV/−30 mV is used as the stability standard [33]. However, in non-water-soluble solvent systems such as IPA and DMF used in this experiment, the dielectric constant is significantly lower than that of water. As a result, the DLVO theory is applied differently, and the threshold zeta potential required for stability is lower than in aqueous systems [33]. Furthermore, while this solution contains a siloxane binder formed from MTMS and TEOS, the stability of such sol–gel systems is influenced not only by electrostatic repulsion from particle surface charges, but also by steric hindrance from polymer chains in the solvent. These zeta potential measurements confirmed that stronger deposition is possible depending on the variation in TEOS content and further indicated that an increase in heat resistance can be expected.

### 3.3. Microstructure and Elemental Composition (SEM/EDS)

**Figure 3:**
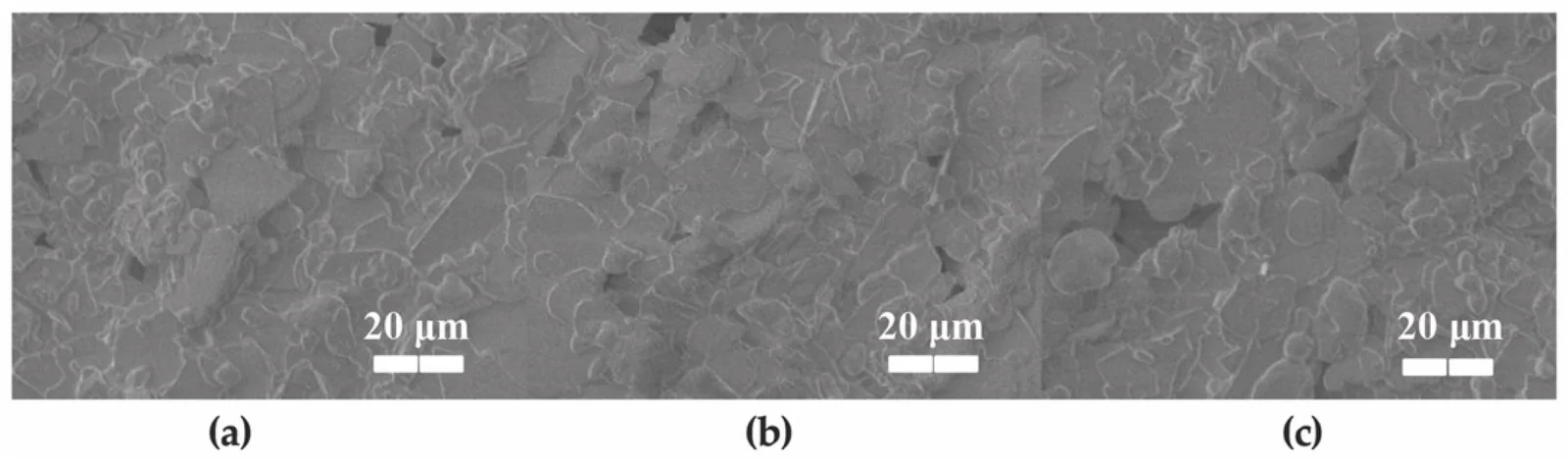
Surface SEM images of specimens with varying TEOS contents: (**a**) TEOS 0 wt%; (**b**) TEOS 10 wt%; (**c**) TEOS 30 wt%.

**Figure 4:**
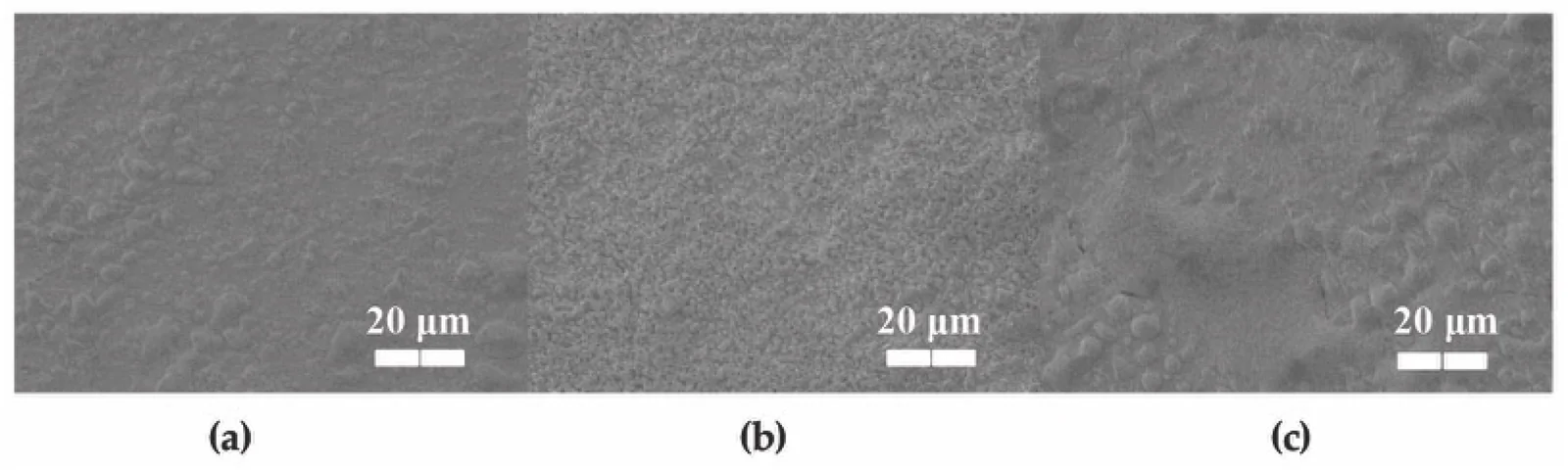
Back-sides SEM images of specimens with varying TEOS contents: (**a**) TEOS 0 wt%; (**b**) TEOS 10 wt%; (**c**) TEOS 30 wt%.

SEM images (Figure 3 and Figure 4) of the front and back surfaces revealed that tabula alumina particles were uniformly deposited on the front surface across all three samples. SEM measurements (Figure 4) and EDS measurements (Table 4) of the back surface indicate the presence of alumina particles, spherical silica particles, and silane.

### 3.4. Thermal Stability and Fire Resistance Performance

**Figure 5:**
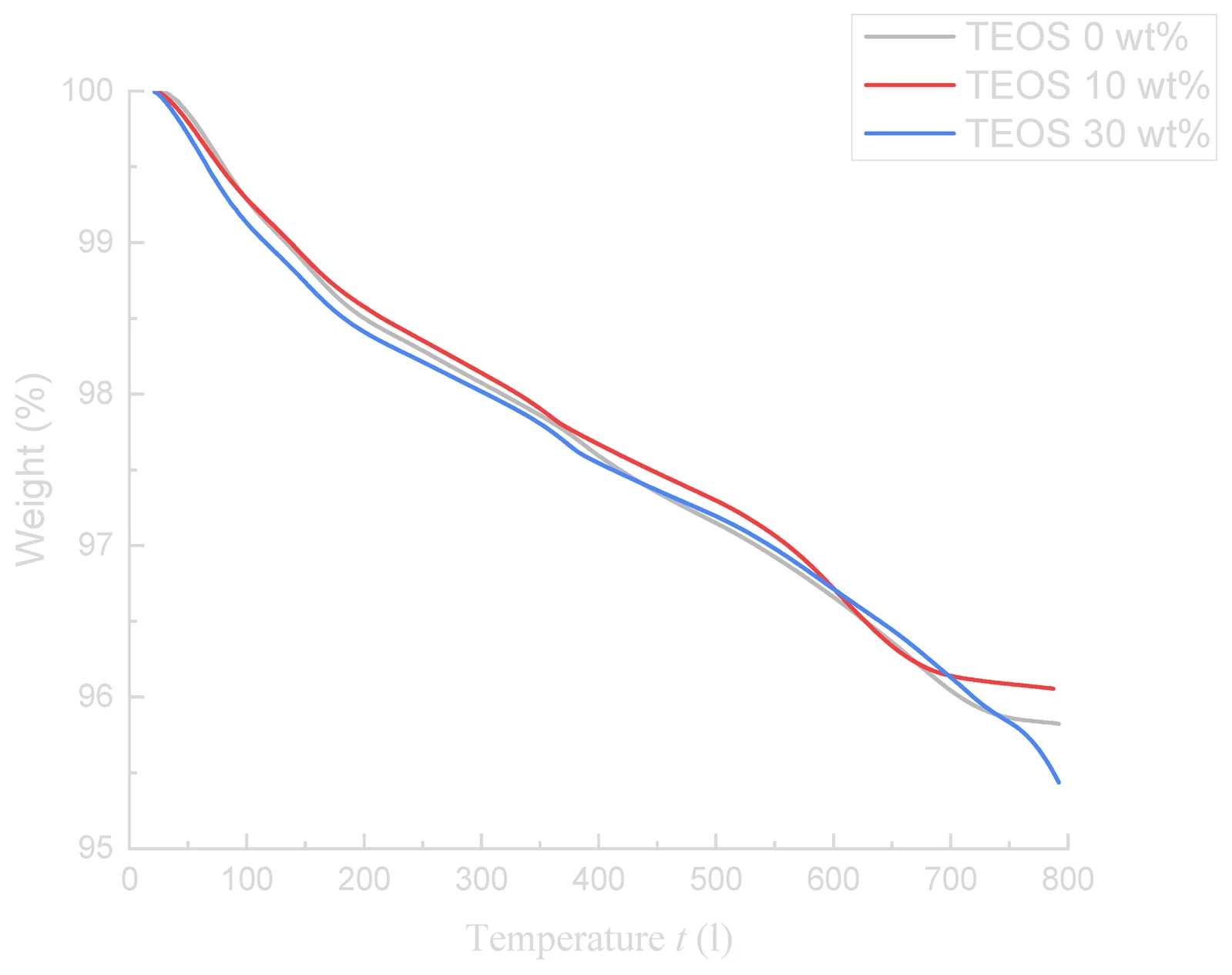
TGA curves of raw materials with different TEOS contents (TEOS 0 wt%, TEOS 10 wt%, TEOS 30 wt%).

TGA analysis was conducted on each sample at a rate of 10 °C per minute, ranging from room temperature to 800 °C. The analysis results (Figure 5) indicate that at least 95% of all samples maintained their temperature at 800 °C during the TGA analysis, regardless of TEOS content. The crossover of the TGA curves observed around 700 °C is attributed to the distinct thermal decomposition kinetics of the binder components; specifically, it stems from the competitive transition between the thermo-oxidative degradation of residual methyl groups in MTMS and the complete ceramization of the TEOS-derived siloxane network, which shifts the respective mass loss rates at elevated temperatures.

As shown in Table 5, in tests simulating actual thermal environments, film detachment occurred the slowest in TEOS 30%, as predicted by prior zeta potential, SEM, and EDS findings. Consequently, TEOS 30% withstood the highest temperature. This suggests that TEOS, compared to MTMS, increases the density of the siloxane network with its tetrafunctional groups, thereby improving mechanical properties and overall thermal stability [29,30].

### 3.5. Dielectric Strength and Insulation Performance

In the case of insulation performance (Table 6), all samples passed the maximum voltage of the insulation tester (6.3 kV), regardless of TEOS content. This confirms that there was no decrease in insulation performance due to TEOS content.

## 4. Discussion

### 4.1. Mechanical Interlocking Effect and Substrate Surface Optimization

The surface condition of the substrate in the EPD coating directly impacts the adhesion of deposited particles and the formation of defects during the drying process. AFM analysis revealed that the average roughness (Sq) of the copper sample pre-treated for 1 min increased to 1.1706 µm, compared to the untreated sample (0.8807 µm). This increase in Sq indicates that the formation of micro-surface irregularities expanded the effective contact area, significantly enhancing the overall bonding strength through the mechanical interlocking effect. When deposition was performed under this optimized roughness, a dense coating layer without cracks was formed, as the improved adhesion effectively suppressed the shrinkage stress generated during the solvent evaporation phase. In contrast, excessive treatment times (over 2 min) or high-temperature conditions (50 °C) resulted in surface dissolution and rearrangement, causing rapid planarization and a decrease in Sq. This reduction in roughness weakened the interlocking mechanism, leading to the formation of cracks, as observed in the untreated specimens.

### 4.2. Non-Linear Gelation Kinetics of T/Q Silane Systems

The failure of the 20 wt% TEOS sample during synthesis, characterized by rapid gelation, is attributed to the complex hybrid sol–gel reaction kinetics between T-type (MTMS) and Q-type (TEOS) silanes. Although increasing the TEOS content generally promotes crosslinking density, the gelation behavior of TQ silanes is non-linear and highly sensitive to the specific T/Q weight ratio. The 20% TEOS ratio appears to represent a critical inflection point where MTMS and TEOS chains crosslink unevenly, resulting in a rapid increase in molecular weight and the shortest gelation time. In contrast, at a 30% TEOS ratio, the system surpasses this critical point, allowing the T-O-Q copolymer to react more uniformly. This results in a longer gelation time compared to the 20% sample, allowing for the stable synthesis of a metastable EPD solution suitable for deposition.

### 4.3. Enhanced Thermal Stability and Siloxane Network Density

The fire resistance tests demonstrated that the 30 wt% TEOS sample withstood the highest temperature of 670 °C, significantly outperforming the 10 wt% (630 °C) and 0 wt% (600 °C) samples. This superior performance is primarily due to the tetrafunctional nature of TEOS compared to the trifunctional MTMS. Increasing the TEOS content enhanced the density of the siloxane (Si-O-Si) network, resulting in a more robust and rigid inorganic framework. This high-density structure effectively resists thermal degradation and maintains the mechanical integrity of the coating even under extreme heat, which is crucial for protecting EV busbars during thermal runaway events. Furthermore, the zeta potential analysis revealed that the 30% TEOS sample had the highest absolute value (−15.5 mV), indicating that improved colloidal dispersion stability also contributed to the formation of a more uniform and defect-free protective layer.

### 4.4. Dielectric Reliability of Composite Coatings

Despite the variations in TEOS content and the resulting changes in thermal properties, all samples successfully passed the dielectric strength test at the maximum voltage of 6.3 kV without any pinhole defects. This confirms that the insulation performance is primarily upheld by the alumina–silica hybrid structure and remains unaffected by the increase in the TEOS weight ratio. These findings demonstrate that the proposed EPD coating strategy effectively balances high-temperature fire resistance with the high-voltage insulation requirements essential for EV power transmission components.

## 5. Conclusions

This study aimed to develop an alumina–silica composite insulating and heat-resistant coating using electrophoretic deposition (EPD) to enhance the stability of electrode materials during electric vehicle (EV) fires. To ensure the quality of the EPD process, we first evaluated the coating properties based on the surface roughness of the copper electrode material. Subsequently, experiments were conducted by varying the weight ratio of MTMS to TEOS from 0% to 30% to optimize the composition of the siloxane binder. AFM analysis revealed that samples pre-treated with the coating solution for 1 min exhibited higher surface roughness compared to the untreated samples, confirming that this treatment suppressed cracks generated during the drying process after coating. This supports the notion that increasing the surface area of the substrate in the EPD process enhances the bonding strength of the coating.

During the preparation of the coating solution with the MTMS/TEOS weight ratio, the 20% TEOS sample underwent rapid gelation, rendering EPD deposition impossible. This rapid gelation is attributed to the increased network formation rate resulting from the high reactivity of the tetrafunctional TEOS. Comparative analysis of the 0%, 10%, and 30% samples revealed that TGA analysis indicated that all samples retained over 95% of their original volume even at 800 °C, confirming excellent basic heat resistance. Insulation performance was validated through pinhole testing, where all samples successfully withstood a maximum voltage of 6.3 kV, demonstrating that the TEOS content did not adversely affect insulation performance. In a crucial ignition test, the 30% TEOS sample exhibited the best heat resistance at 670 °C, representing a significant improvement compared to the 10% TEOS (630 °C) and 0% TEOS (600 °C) samples. Zeta potential analysis indicated the highest absolute value for the 30% TEOS sample (−15.5 mV), suggesting a slight enhancement in the metastable phase state and dispersion stability in solution. In conclusion, increasing the Q-type (tetrafunctional) TEOS content to 30% relative to T-type (trifunctional) MTMS enhanced the density of the siloxane network, improving the mechanical properties. This led to improved overall structural and thermal stability of the coating. Therefore, adjusting the TEOS content in alumina–silica composite coatings using the EPD method has been confirmed as an effective strategy for ensuring the fire safety of electrode materials in electric vehicles.

## Figures and Tables

**Table 1:** Composition of the TEOS/MTMS silane binders by weight ratio.

Sample	MTMS (g)	TEOS (g)	PS-30 (g)	TM-50 (g)	TEOS Weight Ratio Relative to Total Silane
TEOS 0 wt%	188 g	0 g	80 g	165 g	0%
TEOS 10 wt%	169.2 g	18.8 g	80 g	165 g	10%
TEOS 20 wt%	150.4 g	37.6 g	80 g	165 g	20%
TEOS 30 wt%	131.6 g	56.4 g	80 g	165 g	30%

**Table 2:** Average roughness according to surface treatment.

Sample	Sq (µm)
Untreated sample	0.8807
1 min	1.1706
2 min	0.6523
30 °C 1 min	0.5475
50 °C 1 min	0.8757

**Table 3:** Zeta potential analysis of specimen according to TEOS content.

Sample	Zeta Potential (mV)
TEOS 0 wt%	−3.63
TEOS 10 wt%	−11.2
TEOS 30 wt%	−15.5

**Table 4:** EDS analysis of specimen back-sides as a function of TEOS content.

Sample	Al Content (Mass%)	Si Content (Mass%)
TEOS 0 wt%	9.22	90.78
TEOS 10 wt%	9.19	90.81
TEOS 30 wt%	3.09	97.03

**Table 5:** Results of actual fire environment tests according to TEOS content.

Sample	Actual Fire Environment Tests
TEOS 0 wt%	600 °C
TEOS 10 wt%	630 °C
TEOS 30 wt%	670 °C

**Table 6:** Results of pinhole tests according to TEOS content.

Sample	Pinhole Tests
TEOS 0 wt%	6.3 kV
TEOS 10 wt%	6.3 kV
TEOS 30 wt%	6.3 kV

## Data Availability

The original contributions presented in this study are included in the article. Further inquiries can be directed to the corresponding author.
